# A rapid beam simulation framework for transcranial focused ultrasound

**DOI:** 10.1038/s41598-019-43775-6

**Published:** 2019-05-28

**Authors:** Steven A. Leung, Taylor D. Webb, Rachelle R. Bitton, Pejman Ghanouni, Kim Butts Pauly

**Affiliations:** 1 Department of Bioengineering, Stanford University, Stanford, USA; 2 Department of Electrical Engineering, Stanford University, Stanford, USA; 3 Department of Radiology, Stanford University, Stanford, USA

**Keywords:** Movement disorders, Magnetic resonance imaging, Computed tomography, Therapeutics, Biomedical engineering

## Abstract

Transcranial focused ultrasound is a non-invasive therapeutic modality that can be used to treat essential tremor. Beams of energy are focused into a small spot in the thalamus, resulting in tissue heating and ablation. Here, we report on a rapid 3D numeric simulation framework that can be used to predict focal spot characteristics prior to the application of ultrasound. By comparing with magnetic resonance proton resonance frequency shift thermometry (MR thermometry) data acquired during treatments of essential tremor, we verified that our simulation framework can be used to predict focal spot position, and with patient-specific calibration, predict focal spot temperature rise. Preliminary data suggests that lateral smearing of the focal spot can be simulated. The framework may also be relevant for other therapeutic ultrasound applications such as blood brain barrier opening and neuromodulation.

## Introduction

Transcranial focused ultrasound (FUS) is a therapeutic modality that can be used to induce transient or permanent changes in brain tissue. The most common applications include ablation^1–3^, blood brain barrier opening^4–7^, and neuromodulation^8–10^.

**Figure 1:**
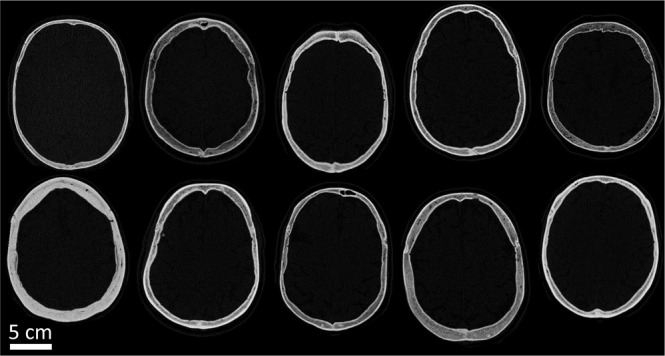
Computed tomography images of patient skulls demonstrate heterogeneity across patients. There is variability in size, thickness, shape, and proportion of cortical and trabecular bone within and across patients.

For all transcranial applications, the ultrasound energy must be focused through the skull. However, the skull varies within and across patients in terms of size, thickness, shape, and proportion of cortical to trabecular bone (Fig. 1). Therefore, as ultrasound propagates through the skull, the bone heterogeneously reflects, refracts, and attenuates the ultrasound waves, thus altering the position, intensity, and shape of the beam’s focus. The complex interactions with the skull cannot be perfectly determined through analytical means, therefore non-invasive measurements are used to evaluate focal spot characteristics. During ablation treatments, magnetic resonance proton resonance frequency shift thermometry (MR thermometry) measures temperature rise due to FUS in a single cross section of the brain. Measuring the temperature rise is important because there is a broad range in skull efficiencies across patients^11^, such that the same applied power can result in a four-fold range in focal spot temperature rise.

In current clinical practice, the focal spot position and temperature rise for a given set of parameters are unknown prior to treatment. Therefore, at the initiation of a treatment, the focal spot must be characterized to confirm targeting and the safety of proceeding with treatment. Multiple sonications are delivered for iterative calibration, using energies adequate to create a reliably measurable temperature rise but considered insufficient for permanent neural ablation. As part of this iterative process, element phases and amplitudes can be modified until the desired focal spot is achieved. Safety may be improved by achieving the desired temperature rise and by reducing the number of sonications needed for calibration. Both actions reduce the amount of unwanted heating in the brain. In addition, experience has revealed that energy delivery across the skull becomes progressively more difficult as the number of sonications increase; this may be due to a change in the properties of the brain tissue^12^ or the skull^13^ as treatment continues (Supplementary Fig. S3a). This phenomenon results in the use of higher energies to achieve the desired temperature rise sufficient for ablation and durable symptom relief. In order to avoid this issue, practitioners now attempt to ramp energies to achieve ablative temperatures rapidly, in as few sonications as possible, while maintaining safety. Simulations that accurately predict the parameters needed to produce a focal spot at a desired location and of a given size could result in fewer sonications during treatment, thereby shortening treatment duration and potentially improving efficacy and safety. Such simulations are also relevant for non-heating applications such as blood brain barrier opening and neuromodulation, wherein there is no temperature rise to be measured and an alternative method is needed to perform focal spot characterization.

In previous studies, 3D numeric simulations have been used for predicting focal spot characteristics. In general, numeric simulation frameworks use patient-specific skull models to account for heterogeneities of the skull. These models are derived from computed tomography (CT) images of individual patients. For every voxel in a skull model, acoustic properties can be assigned according to its CT Hounsfield unit (HU) value. To simulate the ultrasound, many frameworks solve second order partial differential wave and/or elastic equations using a finite difference time domain (FDTD) method^14–25^, a pseudo spectral time domain (PSTD) method^26–28^, or a hybrid angular spectrum (HAS) method^11,29–31^.

Here, we propose a rapid 3D numeric simulation framework that uses the HAS method to predict focal spot characteristics. We designed our simulations to be treatment-specific; in addition to using patient-specific skull models, we accounted for the exact patient position during treatment and the element phases and amplitudes used during each sonication. Only one study to date has been treatment-specific^21^, though it simulated many fewer sonications and did not use the HAS method. The HAS method is two orders of magnitude faster than FDTD and PSTD methods^29^, reducing computation time from tens of hours to minutes. With its advantage in speed, HAS opens the possibility for performing real-time simulation of treatment and for rapidly adjusting to treatment modifications, such as repositioning of the target to adequately relieve symptoms or to avoid side effects, or the application of transducer apodization to selectively reduce energy delivery from certain elements. To evaluate the accuracy of our simulation framework, we compared our simulation results with MR thermometry data from a unique clinical dataset acquired during treatment of patients with essential tremor.

## Results

**Figure 2:**
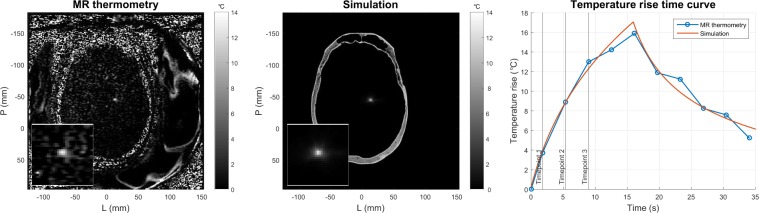
MR thermometry and simulation focal spots for a single sonication (patient D). The simulated temperature rise time curve was calculated using the bioheat equation.

Focal spot position and temperature rise were compared between MR thermometry and simulation. An example of the focal spots and their corresponding temperature rise time curves is shown in Fig. 2. Simulations took three minutes to compute on seven CPU cores (Intel Xeon E5-2640v4 2.40 GHz processor) running in parallel. Each CPU core was allocated 12 GB of memory. The simulation framework was not trained on the data; no parameter tuning was performed to improve the results. Acoustic properties and bioheat tissue properties were referenced from the literature and design decisions were made to improve interpretability and repeatability of results.

### Position

We defined the focal spot position to be at the pixel with the highest temperature rise at the 3^rd^ MR thermometry image (timepoint 3). Position error was calculated as the distance between the MR thermometry and simulation focal spots. The error was separately calculated in the frequency and phase encode directions to separate the effects of off-resonance shift and different pixel sizes. Position errors were averaged per patient and across all patients.

**Figure 3:**
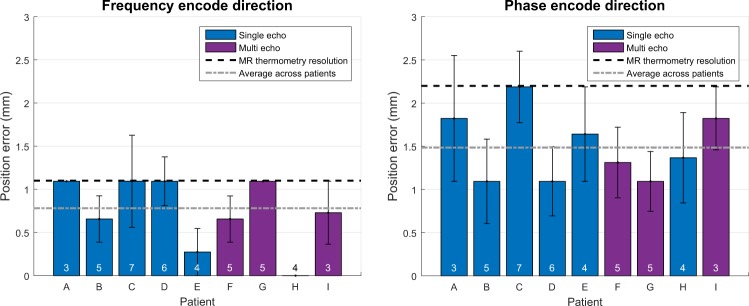
Performance of HAS simulations based on focal spot position. The average position error per patient is shown. Standard error bars are included. Single echo and multi echo thermometry data are denoted in blue and purple, respectively. The number of included sonications per patient is indicated at the bottom of each bar.

Position errors averaged per patient are shown in Fig. 3. The mean position error across patients was 0.781 mm and 1.484 mm in the frequency and phase encode directions, respectively. The position error was not significantly different between single echo and multi echo data in either the frequency encode direction (p = 0.963) or the phase encode direction (p = 0.483). Electronic steering had no pronounced effect on position error (Supplementary Fig. S1). During a given treatment, the focal spots were electronically steered by a mean distance of 1.566 mm and a maximum distance of 2.588 mm from the geometric focus.

### Temperature rise

**Figure 4:**
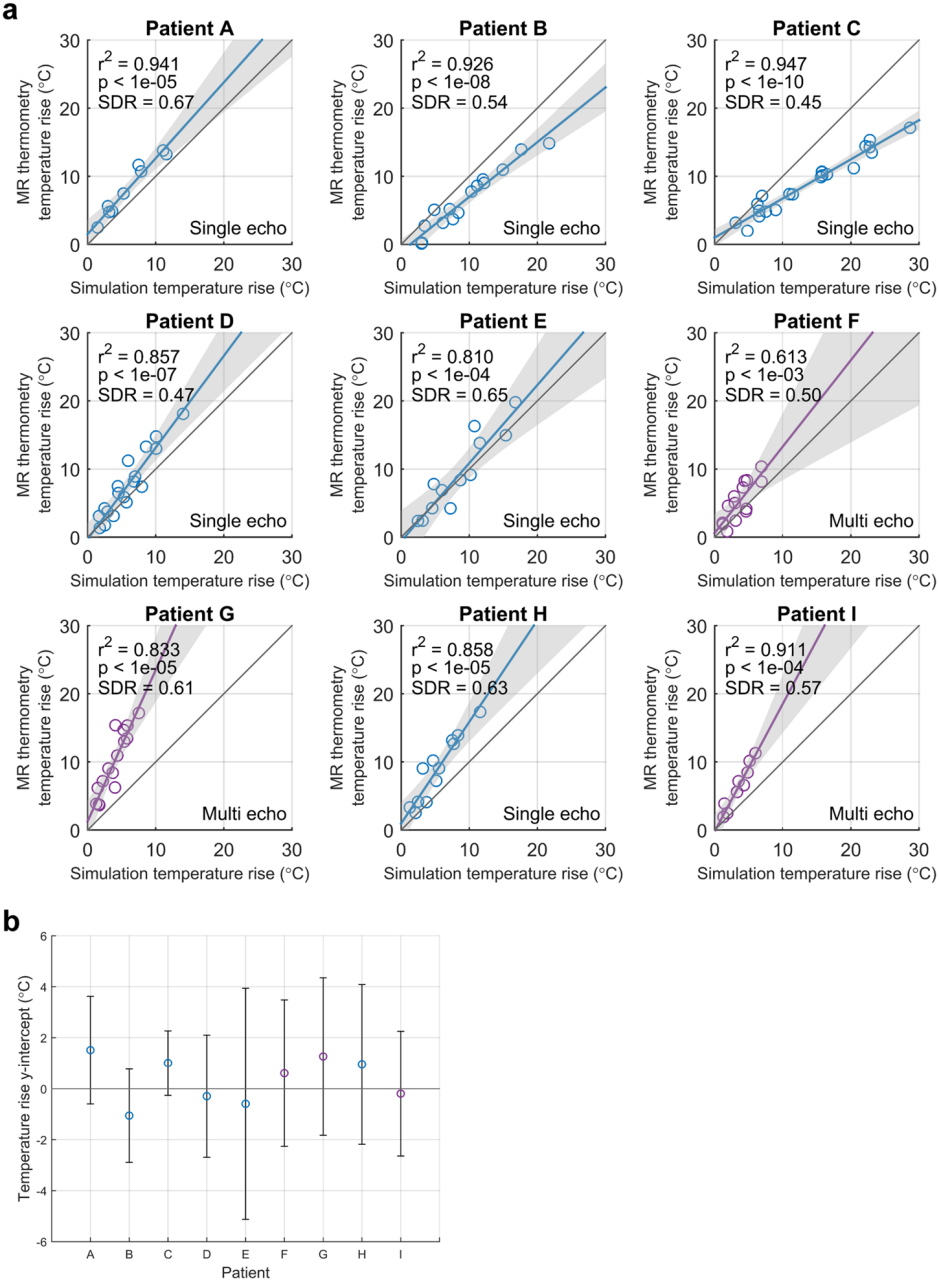
Performance of HAS simulations based on focal spot temperature rise. (**a**) The MR thermometry and simulation peak temperature rises at timepoints 1–3 are plotted. The unity line is in gray. Single echo and multi echo thermometry data are denoted in blue and purple, respectively. Robust linear regressions are plotted with their 95% confidence intervals shaded in gray. Adjusted r^2^ values, p values, and SDRs are also reported. (**b**) The y-intercepts and 95% confidence intervals for each patient are shown.

Robust linear regressions were performed per patient between MR thermometry and simulation peak temperature rise. Temperature rises at timepoints 1–3 were used for comparison. The same focal spot position (previously determined to calculate the position error) was used to evaluate temperature rise at all three timepoints. The linear regressions and their 95% confidence intervals are shown in Fig. 4a. The corresponding y-intercepts and their 95% confidence intervals are shown in Fig. 4b. Per patient, the simulation temperature rise had a statistically significant linear association with the MR thermometry temperature rise (p < 0.05). Age, size of skull, and skull density ratio (SDR) were not predictors of whether simulation overestimated or underestimated temperature rise (Supplementary Fig. S2). SDR is a heuristic for the heterogeneity in a skull. It is calculated by drawing a line between each transducer element and the geometric focus, then taking the ratio between the minimum and maximum HU values along each line. The ratios are averaged to yield the SDR. Low values of SDR indicate high patient bone heterogeneity; high values of SDR indicate low heterogeneity.

**Figure 5:**
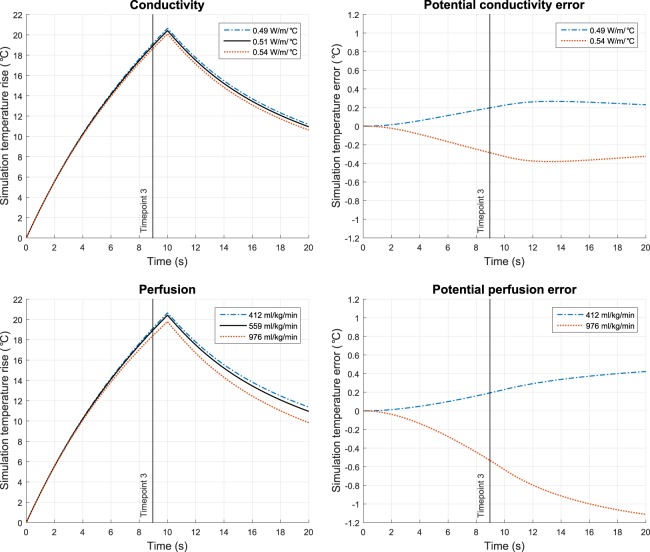
Simulation of temperature rise with variability in the conductivity and perfusion brain tissue properties. Plausible values of conductivity and perfusion resulted in the depicted variation in temperature rise. The range of values used were reported by IT’IS^32^.

Simulated temperature rise can be affected by the selection of bioheat brain tissue properties. To evaluate the magnitude of potential error due to variability in tissue properties, we tested the entire range of brain tissue conductivity (0.49–0.54 W/m/°C) and perfusion (412–976 ml/kg/min) values reported in literature^32^. Conductivity and perfusion were the two properties with the largest percent difference range in values. For a 19 °C temperature rise at timepoint 3, the conductivity and perfusion values resulted in a 0.48 °C and a 0.73 °C difference in temperature rise, respectively (Fig. 5).

**Figure 6:**
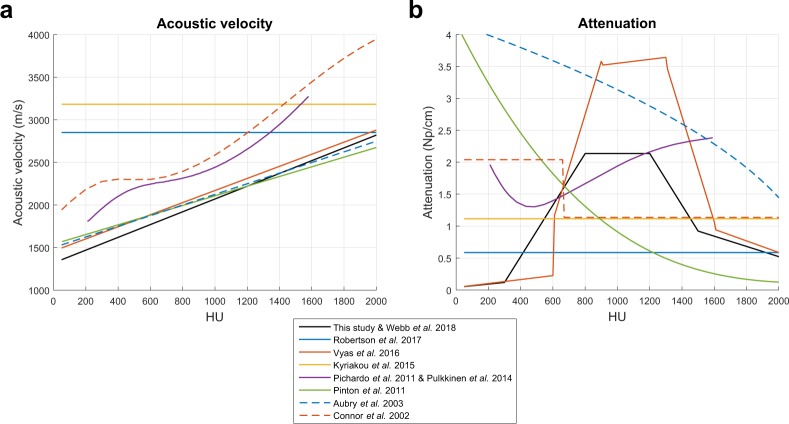
Uncertainty in the human skull acoustic properties. (**a**) There is some consensus in the literature as to the underlying relationship between acoustic velocity and HU. (**b**) There is no consensus in the literature as to the underlying relationship between attenuation and HU. The attenuation values were scaled to 680 kHz using a linear scaling factor.

**Figure 7:**
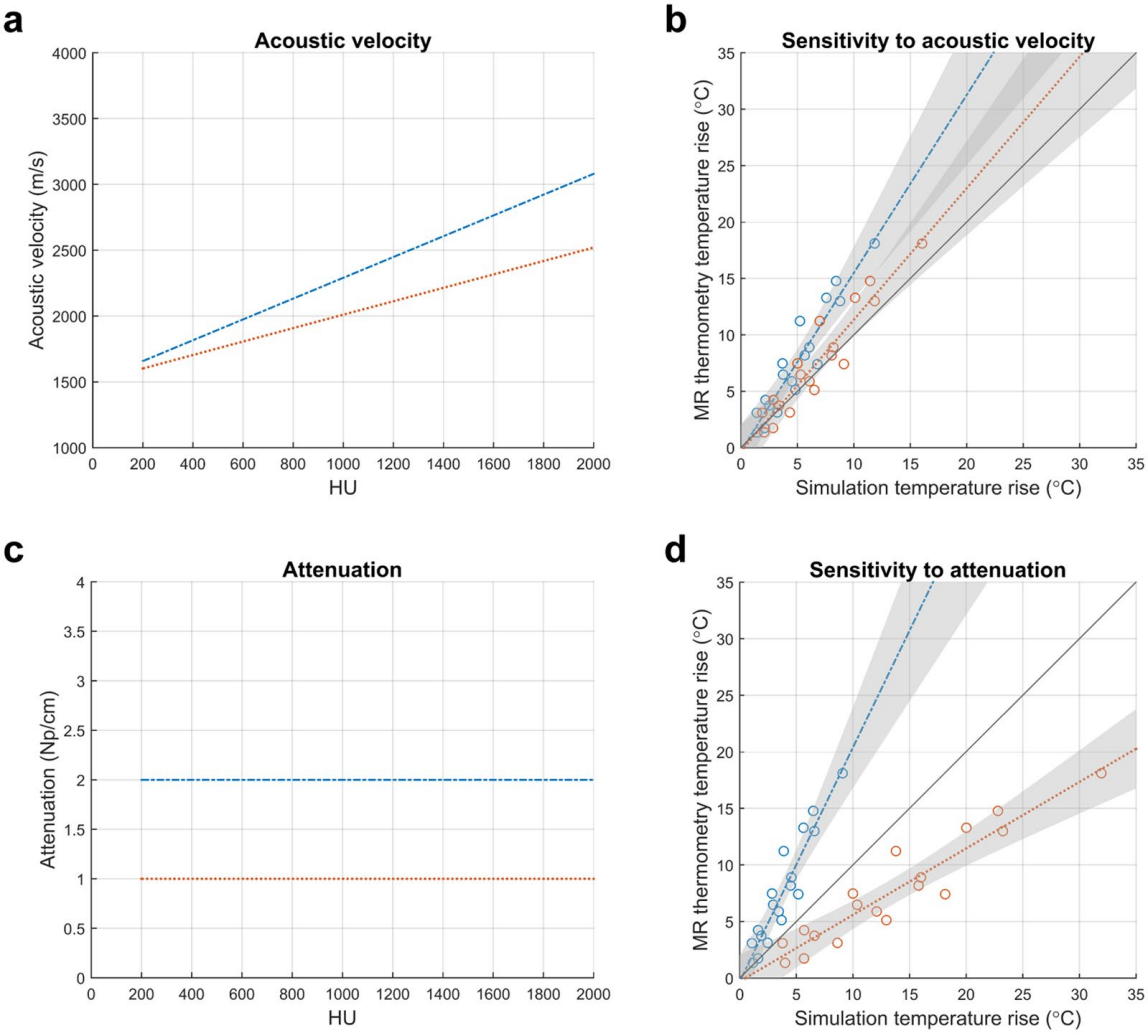
The effects of skull acoustic properties on simulated temperature rise (patient D). (**a**) Acoustic velocity curves as a function of HU. The maximum acoustic velocities at 2000 HU are ±10% of the maximum acoustic velocity used in this study. (**b**) Simulated temperature rises that resulted from using the acoustic velocity curves in (**a**). (**c**) Constant attenuation curves as a function of HU. The two constants are within the plausible range of attenuation values (Fig. 6b). (**d**) Simulated temperature rises that resulted from using the attenuation curves in (**c**).

Simulated temperature rise can also be affected by the selection of skull acoustic property relationships. The underlying relationships between human skull acoustic velocity and attenuation as functions of HU are not well understood, as seen in the literature (Fig. 6). To illustrate the influence of acoustic velocity on simulated temperature rise, we modeled two curves with y-intercepts at 1500 m/s and maximum acoustic velocities of 2520 m/s and 3080 m/s at 2000 HU (Fig. 7a). These values were ±10% of the maximum acoustic velocity used in this study. The two acoustic velocity curves resulted in an approximate 1.3-fold range in simulated temperature rise (Fig. 7b). To illustrate the influence of attenuation on simulated temperature rise, we modeled two constant attenuation curves (Fig. 7c) in the plausible range of attenuation values (Fig. 6b). Lower total attenuation (orange dotted line) resulted in higher simulated temperature rises. Changing the total amount of attenuation affected the slope of the linear regression. These two attenuation curves (1 Np/cm and 2 Np/cm) had differences smaller than the variability depicted in Fig. 6b and resulted in an approximate four-fold range in simulated temperature rise (Fig. 7d).

### Shape

**Figure 8:**
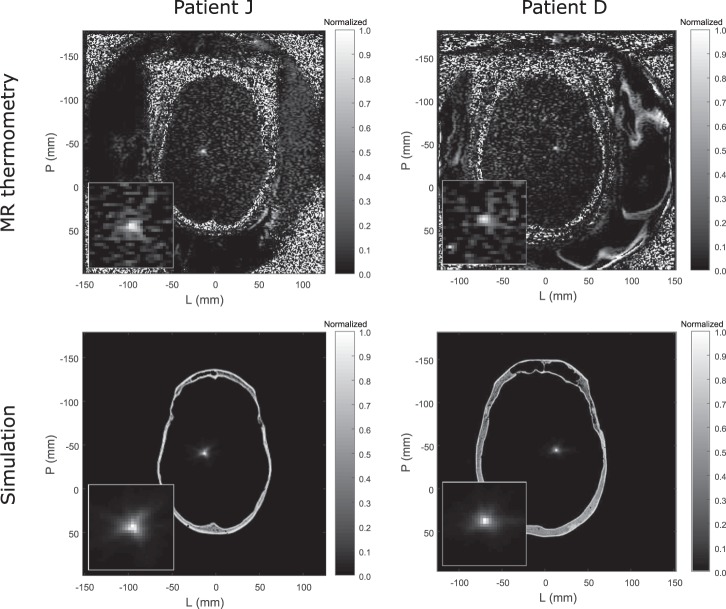
Comparisons of MR thermometry and simulation focal spot shape (patients J and D). The bottom left inset shows an enlarged portion of the image. Lateral smearing in the axial plane can be seen for patient J in both MR thermometry and simulation. Lateral smearing did not occur for patient D.

In both MR thermometry and simulation, all nine patients had circularly shaped focal spots in the axial plane. An additional patient, patient J, had an irregularly shaped focal spot that was observed during treatment (Fig. 8 top left panel). This patient had thin temporal bone, which led to lower attenuation of energy from the lateral transducer elements and resulted in lateral smearing of the focal spot. The lateral smearing is shown in both MR thermometry and simulation (Fig. 8 left column). Patient D (Fig. 8 right column) is shown for comparison to contrast a circularly shaped focal spot with the laterally smeared focal spot. Patient J was not included in the position and temperature rise analysis because the patient’s CT images did not satisfy this study’s CT image acquisition constraints.

## Discussion

In this study, we directly compared the MR thermometry and simulation focal spots to evaluate the accuracy of the HAS framework. The simulation framework performs well at predicting focal spot position. Predicted temperature rise has multiplicative error, which can be accounted for after patient-specific calibration. There is also preliminary evidence for successful shape prediction. With its ability to rapidly predict focal spot characteristics, the HAS framework has great potential for use in patient screening, pre-treatment planning, and decision support. The framework can also be extended for use in applications of blood brain barrier opening and neuromodulation.

### Position

The simulation performed well at predicting focal spot position. The 0.781 mm and 1.484 mm errors in the frequency and phase encode directions were less than the MR thermometry resolution. This suggests that our acoustic velocity model is sufficiently accurate for the resolution at which we performed the simulations. Additionally, both single echo and multi echo data can be used for comparison with simulation, as there was no statistical significance between the two in either the frequency and phase encode directions.

Future work is needed to simulate treatments in which the intended focal spots are far away from the transducer’s geometric focus. Such work will determine the simulation envelope in which focal spot position can be accurately predicted. The focal spots analyzed in this study were relatively close to the geometric focus; they were electronically steered by a mean distance of 1.566 mm and a maximum distance of 2.588 mm. This range of electronic steering is relatively small compared to the treatment envelope, which has a radius of approximately 15–20 mm at 680 kHz^33^, though much of the treatment envelope extent is due to mechanical steering. Phantom experiments will likely be needed to test this simulation envelope, because essential tremor treatments targeting the ventral intermediate nucleus will typically not require electronic steering of substantial distances.

### Temperature rise

The robust linear regression models show the HAS framework’s consistency in simulating temperature rise. Even though electronic steering resulted in modifications to the element phases and amplitudes, the simulation temperature rise maintained a statistically significant linear association with the MR thermometry temperature rise (p < 0.05). In addition, all of the linear models contained the origin in the 95% confidence intervals of their y-intercepts, demonstrating the expected behavior that no MR thermometry temperature rise is present when there is no simulated temperature rise. The linear models have non-unity slope, which results in multiplicative error in the predicted temperature rise. With several patient-specific calibration sonications, the slope can be determined and then used to predict temperature rise of future sonications, even while using new sets of element phases and amplitudes.

Ideally, the ultimate goal of this simulation framework would be to produce unity slope for all patients. To better understand the possible sources of temperature rise error, we investigated several individual components of the simulation framework. Error could originate from the HAS method, which has two main shortcomings. First, the HAS method does not simulate nonlinearity of ultrasound. This is due to the method’s assumption of a monochromatic ultrasound source at steady state. For 1 MHz ultrasound, modeling nonlinearity could result in up to a 2% increase in simulated temperature rise at 2 MPa^34^, 13.1% increase at 4 MPa^35^, and 27% increase at 5 MPa^34^. Because attenuation is frequency dependent, we expect smaller errors due to nonlinearity at lower frequencies of ultrasound. For 670 kHz ultrasound, Sapozhnikov *et al*. reported that 10% of wave power is transferred to the higher harmonics at tens of MPa^36^. Across all patient sonications that were simulated in this study, the maximum pressure was 4.5 MPa while most sonications were in the 2–3 MPa range. These values corresponded well with the values reported by Pulkkinen *et al*. For blood brain barrier opening and neuromodulation applications, even less acoustic power is applied, therefore we would expect even smaller effects of nonlinearity. Second, the HAS method does not account for mode conversion from longitudinal to shear waves. This is due to the method’s derivation from the scalar wave equation rather than the visco-elastic wave equation. Modeling mode conversion could result in a 2.81%^28^ or 9%^21^ decrease in simulated temperature rise. Nevertheless, neither nonlinearity nor mode conversion alone are substantial enough to explain the errors in simulated temperature rise observed in the results.

Additional sources of error could be inaccurate assumptions about the thermal properties of brain tissue. Choosing the incorrect property values for the bioheat equation will result in systematic underestimation or overestimation of temperature rise. For a 19 °C temperature rise at timepoint 3, the entire range of brain tissue conductivity and perfusion values resulted in a 0.48 °C and a 0.73 °C difference in temperature rise, respectively (Fig. 5). These variations were not substantial enough to explain the temperature rise underestimation in patients G and I nor overestimation in patients B and C. Therefore, variability in conductivity or perfusion did not explain the errors in simulated temperature rise.

Further sources of error could arise from inaccurate assumptions about the acoustic properties of the human skull. As seen in Fig. 6, there is uncertainty in the literature as to the relationships of acoustic velocity and attenuation as functions of HU. For this study, we simulated only treatments for which the CT images were acquired on a General Electric CT scanner using 120 kVp tube voltage, MEDIUM filter, and BONEPLUS reconstruction kernel. This eliminated several sources of variability from the CT acquisition, which we see from Webb *et al*. would result in a shift in acoustic velocity as a function of HU. In the absence of this CT acquisition variability, some uncertainty in the acoustic velocity may still be present, but the literature is surprisingly consistent: Fig. 6a shows several of the curves following the same relationship (Aubry *et al*., Pinton *et al*., and Vyas *et al*.). There are two other sets of curves that appear to be in different regimes, and the differences may be because unknown CT tube voltage was used (Connor *et al*. and Pichardo *et al*.) or because the simulations were not performed with CT images (Kyriakou *et al*. and Robertson *et al*.). In Fig. 7a,b, we consider the impact of ±10% error in acoustic velocity at 2000 HU and find that the two acoustic velocity curves result in an approximate 1.3-fold range in simulated temperature rise. ±10% error in maximum acoustic velocity is greater than the variability seen across Aubry *et al*., Pinton *et al*., and Vyas *et al*., and thus represents the worse case scenario in acoustic velocity error. From this analysis, we see that uncertainty in acoustic velocity is a likely contributor to error in simulated temperature rise but is unlikely to be the main contributor.

Amongst the acoustic properties, attenuation of the skull contains the most uncertainty, as seen by the lack of consensus in the literature (Fig. 6b); the true relationship between human skull attenuation and HU is still not well understood, and is currently being studied. As shown in Fig. 7c,d, the two constant attenuation curves (1 Np/cm and 2 Np/cm) have differences smaller than the variability depicted in Fig. 6b yet result in an approximate four-fold range in simulated temperature rise. Therefore, the attenuation property substantially dictates the relationship between MR thermometry and simulation temperature rise. We hypothesize that the inaccurate modeling of the attenuation property is the main source of error in simulated temperature rise, thus resulting in temperature rise plots with non-unity slopes.

In addition to uncertainty in the attenuation curve, it is unclear whether the curve should be universal across patients or unique for each patient. In this study, and in many others, a universal attenuation curve is assumed. In conjunction with our use of a universal attenuation curve, we reduced simulation variability across patients by using only CTs that satisfied a specific set of acquisition parameters (General Electric CT scanner, 120 kVp tube voltage, MEDIUM filter, and BONEPLUS reconstruction kernel). This established a one-to-one relationship between attenuation and HU. However, there was still variability across patients (Fig. 4) that we did not fully account for, suggesting the plausibility of patient-dependent acoustic properties of bone^37^.

Because the attenuation property greatly affects simulated temperature rise, and the relationship between attenuation and HU is largely unknown, this motivates important future work in determining the underlying relationship. Removing uncertainty in the attenuation model will refine the accuracy in temperature rise prediction. Accurate modeling of the attenuation-HU relationship will also result in improved intensity prediction, which will be useful for non-heating applications such as blood brain barrier opening and neuromodulation where there is no temperature rise that can be used for focal spot characterization.

### Shape

Because the InSightec hemispherical phased array transducer has an f-number of 1 and is highly focused, we expect to see circularly shaped focal spots in the axial plane. This was observed in both MR thermometry and simulation (Fig. 8 right column), where patient D was shown as a representative example. Patient J was an exception; the patient’s thin temporal bone resulted in lateral smearing of the focal spot. Nevertheless, the MR thermometry and simulation focal spots corresponded well with one another for patient J as well (Fig. 8 left column). In addition, the simulation focal spots of patients J and D (Fig. 8 bottom row) are visually distinct in shape, thus providing potentially clinically relevant information regarding lateral smearing of the focal spot.

### Limitations of MR thermometry validation

Using the MR thermometry images for validation had two main limitations: resolution and noise. Although the simulations were performed with sub-millimeter voxel dimensions, they had to be downsampled to match the 1.094 × 2.188 × 3 mm dimensions of the MR thermometry images. This quantized the position error to multiples of 1.094 mm. It is unclear whether the quantization increased or decreased the position error overall. Additionally, image noise and spurious artifacts degraded the accuracy of the position and temperature rise metrics. Shape metrics such as eccentricity and orientation were also found to be highly susceptible to noise at the resolution of the MR thermometry images. Datasets with better resolution and less noise, such as hydrophone measurements, will be a different option for validating simulation focal spot characteristics, though this would preclude *in vivo* studies.

### Exclusion of sonications in the temperature rise roll-off region

Sonications that fell beyond three standard deviations of noise from the treatment efficiency line were excluded because they did not satisfy the expected linear behavior between temperature rise and power applied (Supplementary Fig. S3a). Excluded sonications in this temperature rise roll-off region were consistently later sonications, suggesting that changes in tissue or skull properties had occurred over the course of treatment^12,13^. We did not model changes in tissue or skull properties, therefore we did not include these sonications in this study.

## Conclusion

The hybrid angular spectrum (HAS) framework can be used to predict a focal spot’s position prior to application of ultrasound. With patient-specific calibration, the HAS framework can also predict focal spot temperature rise. In addition, preliminary data suggests that lateral smearing of the focal spot can also be simulated. These conclusions were shown through comparisons with ground truth MR thermometry data. The HAS method’s low computation requirements (CPUs instead of GPUs) make it accessible to many potential users. Its advantage in computation speed allows it to be used in real-time. There is immense potential for the HAS method to be used as a pre-clinical or clinical tool that is robust for experiment design, patient screening, pre-treatment planning, and decision support. The framework may also be applied to applications of blood brain barrier opening and neuromodulation.

## Methods

### MR thermometry data

MR thermometry data were acquired during FUS treatment of patients with essential tremor, in which the ventral intermediate (Vim) nucleus of the thalamus was the primary target. All experimental protocols were approved by the Stanford Investigational Review Board. All methods were carried out with the relevant guidelines and regulations. Informed consent was obtained from all participants in the study.

Single echo and multi echo MR thermometry images were acquired for six patients (A, B, C, D, E, H) and three patients (F, G, I), respectively. The imaging parameters are given in Supplementary Table S1. The thermometry images had dimensions of 256 × 256 pixels, with the resolution being 1.094 × 2.188 mm. The phase encode direction had lower resolution because only 128 lines in the center of k-space were acquired while the outer 128 lines were zero padded. The images were reconstructed with a single baseline, followed by referenceless constant and linear phase term corrections^38^. The thermal coefficient used for proton resonance frequency shift was −0.00909 ppm/°C. A baseline image was acquired for each sonication prior to the acquisition of the 1^st^ MR thermometry image (timepoint 1). Timepoint 1 acquisition and FUS application began at the same time.

We calculated the treatment efficiency (TrEff) metric^39^ and used it to determine which sonications to simulate. TrEff was calculated for each sonication using the 3^rd^ MR thermometry image (timepoint 3):

1 $$Treatment\,efficiency\,(TrEff)=\frac{Temperature\,rise}{Power\,applied}$$

Timepoint 3 was used because it was the latest timepoint at which the ultrasound was on for all sonications (Supplementary Fig. S3b). Because temperature rise continually increases while the ultrasound is on, selecting the latest timepoint for comparison maximizes the SNR. Per patient, the largest two treatment efficiencies were averaged then assigned as the patient’s effective treatment efficiency (TrEff_Effective_). This value was calculated without using alignment sonications (early sonications used to determine the focal spot position in the axial, coronal, and sagittal scan planes). Across all power levels, we included sonications in which the focal spot temperature rise was within three standard deviations of noise from the TrEff_Effective_ line (Supplementary Fig. S3a). Noise standard deviation was individually calculated for each patient; it was measured in a 40 × 40 pixel octagonal frame (frame thickness of 10 pixels) around the focal spot and averaged across all sonications. We excluded sonications beyond three standard deviations from the treatment efficiency line because they no longer satisfied the expected linear increase in temperature rise with respect to power applied^39^. These excluded sonications are referred to as being in the temperature rise roll-off region. We also excluded the first sonication of each treatment. In addition, images that had phase encoding in the superior-inferior direction were excluded to avoid potential artifacts that obscured the focal spot.

### Simulation framework

#### Computational models

We modeled a hemispherical phased array transducer (InSightec ExAblate 4000, InSightec, Tirat Carmel, Israel) for all simulations. The transducer has a diameter of 30 cm, a radius of curvature of 15 cm, 1024 elements, and a fundamental frequency of 680 kHz.

Patient-specific skull models were generated using patient CT images. Individualized skull models are necessary to account for the high degree of skull heterogeneity within and across patients (Fig. 1). To establish consistency across simulations, only CT images acquired on a General Electric (GE) CT scanner using 120 kVp tube voltage, MEDIUM filter, and BONEPLUS reconstruction kernel were used. Because the acoustic property to HU relationships are substantially affected by CT acquisition parameters^37^, these constraints were necessary for model consistency across patients.

In addition to being patient-specific, this study was designed to be treatment-specific. We accounted for the exact positioning of the patients relative to the hemispherical transducer. The registration matrices we used were extracted from the InSightec ExAblate treatment log files. We also accounted for element phases and amplitudes, which were modified from sonication to sonication over the course of a treatment. These phases and amplitudes were applied as simulation parameters.

#### Acoustic properties

The acoustic property relationships are given in Supplementary Table S2 and shown in Supplementary Fig. S4. The relationships are functions of HU. Because we use a standardized set of CT acquisition parameters that affect HU (such as CT tube voltage, filter, and reconstruction kernel), we establish a one-to-one relationship between each acoustic property and HU. Pure cortical bone was defined to have a porosity of 0% and a bone fraction of 1. It has a fixed density (ρ_bone_) and a specific HU value (HU_bone_) at 120 kVp CT tube voltage.

The acoustic velocity relationship used in this study was reported by Webb *et al*.^37^. Bone fraction and density relationships from Vyas *et al*.^11^ were used, though modifications were made to the ρ_bone_ and HU_bone_ constants. Vyas *et al*. used ρ_bone_ = 3000 kg/m^3^ and HU_bone_ = 3115, which resemble properties of hydroxyapatite; we used ρ_bone_ = 1920 kg/m^3^ ^40^ and HU_bone_ = 2000 to better resemble properties of pure cortical bone. HU_bone_ was calculated using ρ_bone_ and mass attenuation coefficients for bone and water reported by the National Institute of Standards and Technology^40^ (Supplementary Fig. S5). We assumed an effective tube voltage of 60 kV for a 120 kVp spectrum because the spectrum data was not available to us.

The porosity and attenuation relationships from Vyas *et al*. were also modified. Vyas *et al*. used a porosity relationship that was patient dependent (stretched or compressed based on a patient’s CT histogram). In this study, we used the same porosity relationship across patients not only to establish a one-to-one relationship between porosity and HU, but also to improve interpretability and repeatability of results. This was possible also because we used a consistent set of CT parameters. We rederived the attenuation curve using empirical studies in bovine femur^41^ and bone-mimicking phantoms^42^ (derivation in supplementary). The major difference in the attenuation curve was the maximum value of the plateau. In Vyas *et al*., the attenuation plateau for trabecular bone was 3.625 Np/cm; in this study, it was 2.136 Np/cm (Supplementary Fig. S6). As a result of this difference, temperature rises simulated using the acoustic properties of Vyas *et al*. will be lower than those simulated in this study.

#### Hybrid angular spectrum (HAS) method

The HAS method was used to propagate each element’s pressure plane through the skull model^43^. Due to the superposition property of ultrasound, the contributions from each element were summed to yield the expected behavior of FUS. The set of pressure planes going through each slice of the skull model constituted the entire simulated volume.

One restriction of the HAS method is the need to simulate pressure planes perpendicular to the direction of propagation. As a result, the InSightec ExAblate head transducer was subdivided into seven individual plates, each of which were used to model separate directions of propagation. The contribution from each individually simulated 3D volume was then summed to yield the resulting focal spot.

#### Bioheat equation

The HAS method computed acoustic pressure, which was then converted to temperature using a finite difference time domain (FDTD) implementation of the bioheat equation^44^:

2 $$\rho C\frac{\partial T}{\partial t}=\kappa {\nabla }^{2}T-{\rho }_{b}{C}_{b}\rho \omega (T-{T}_{b})+\alpha \frac{{P}^{2}}{\rho c}$$

where ρ is the density, C is the specific heat capacity, T is the temperature, t is time, κ is the thermal conductivity, ω is the perfusion rate, α is the attenuation, P is the ultrasound pressure, and c is the acoustic velocity. The subscript b is used to denote blood. The tissue properties used for the bioheat equation are given in Supplementary Table S3.

#### Simulation details

The simulation grid had 1097 × 1097 × 1201 voxels and a voxel size of 0.365 × 0.365 × 0.200 mm (a field of view of 400 × 400 × 240 mm). For each sonication, a 768 × 768 × 15 voxel slab (a field of view of 280 × 280 × 3 mm) was extracted from the simulated volume. The position and orientation of the slab matched the MR thermometry slice position and scan plane. Averaging was performed in-plane and through-plane to yield a 256 × 128 pixel image with 1.094 × 2.188 mm resolution. The image was then upsampled to 256 × 256 pixels to match the dimensions of the MR thermometry image. The underlying resolution of the image remained the same despite the upsampling.

## Supplementary information

Supplementary information: A rapid beam simulation framework for transcranial focused ultrasound

## Data Availability

The data supporting the findings of this study are available within the paper and its Supplementary Information. The MR thermometry data is available from the corresponding author upon reasonable request.

## Code Availability

The simulation framework code is available from the corresponding author upon reasonable request.
