# Supplementary material for: A rapid beam simulation framework for transcranial focused ultrasound
Source: Sci Rep. 2019 May 28;9:7965. doi: 10.1038/s41598-019-43775-6 (PMC6538644; doi:10.1038/s41598-019-43775-6)
Supplement: Supplementary file 1 — Supplementary information: A rapid beam simulation framework for transcranial focused ultrasound [file 41598_2019_43775_MOESM1_ESM.docx]

A rapid beam simulation framework for transcranial focused ultrasound

Steven A. Leung^1^*, Taylor D. Webb^2^, Rachelle R. Bitton^3^, Pejman Ghanouni^3^, Kim Butts Pauly^1,2,3^

# Supplementary Information

**Calculation of HU_bone_**

To justify our use of HU_bone_ = 2000, we show our method for calculating HU_bone_. We performed the calculations using National Institute of Standards and Technology (NIST) reported bone density (ρ_bone_) and mass attenuation coefficients for bone and water [^40^]. We used the standard Hounsfield unit equation:

${HU}_{bone}= 1000* \frac{{\frac{\mu}{\rho}}_{bone}\rho_{bone} - {\frac{\mu}{\rho}}_{water}\rho_{water}}{{\frac{\mu}{\rho}}_{water}\rho_{water} - {\frac{\mu}{\rho}}_{air}\rho_{air}}$ (Equation 1)

where $\frac{\mu}{\rho}$ is the mass attenuation coefficient and ρ is the density. The mass attenuation coefficients of bone and water are functions of photon energy, therefore HU_bone_ is affected by the CT tube voltage. The material densities were ρ_bone_ = 1920 kg/m^3^ and ρ_water_ = 1000 kg/m^3^.

HU_bone_ as a function of effective tube voltage is shown in Supplementary Figure S5. We assumed an effective tube voltage of 60 kV for a 120 kVp spectrum because the spectrum data was not available to us. The resulting HU_bone_ was calculated to be 1965 and rounded up to 2000.

**Derivation of the attenuation model**

We derived the attenuation curve using empirical studies in bovine femur [^41^] and bone-mimicking phantoms [^42^]. The broadband ultrasound attenuation (BUA) was used to calculate the attenuation at 680 kHz (Supplementary Figure S6a). For the Strelitzki et al. curve, the attenuation remained relatively constant for porosities of 44%, 55%, and 60%. Therefore, like Vyas et al., we assumed that attenuation remained constant in the 40-60% porosity range.

Strelitzki et al.’s data was measured on a bone mimicking phantom, which may not necessarily have the same properties as bone. Therefore, we scaled the curve down to be consistent with the values reported by Tavakoli et al., which was 2.136 Np/cm at 40% porosity. To convert porosity to HU, we set 0% to be pure cortical bone (2000 HU) and 100% to be water (0 HU). Connor et al. [^14^] reported the trabecular bone attenuation to be 3 Np/cm/MHz (2.04 Np/cm at 680 kHz), which is in agreement with the Tavakoli et al. reported trabecular bone attenuation of 2.136 Np/cm.


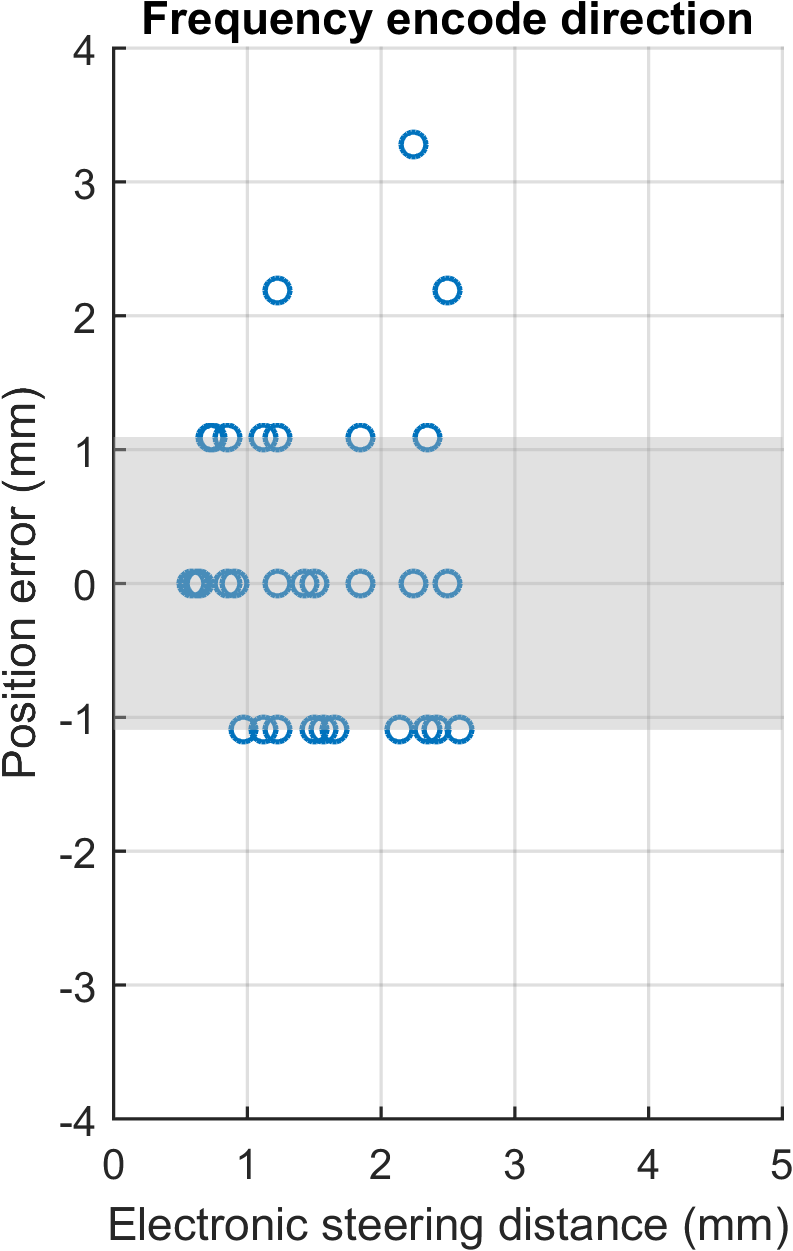

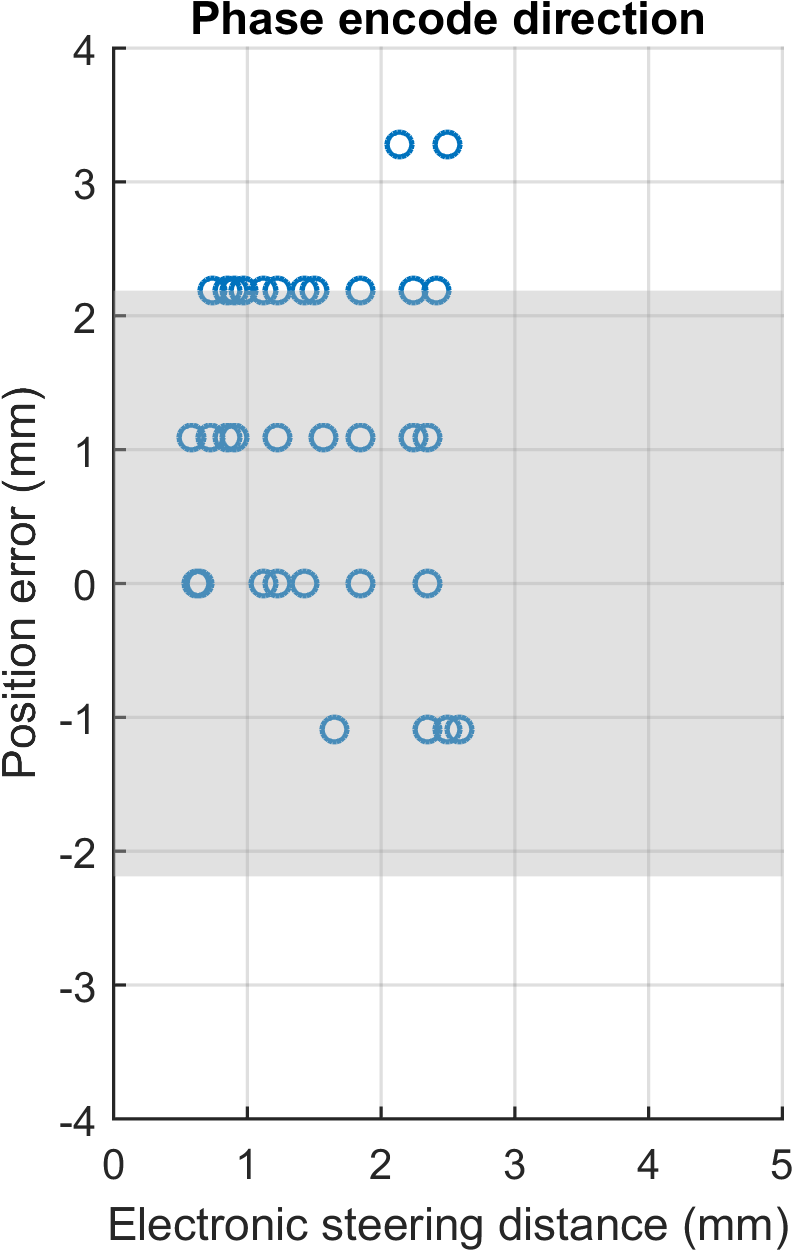


**Supplementary Figure S1.** Position error versus electronic steering distance from the geometric focus. The gray bands delineate the MR thermometry resolution in the frequency and phase encode directions.


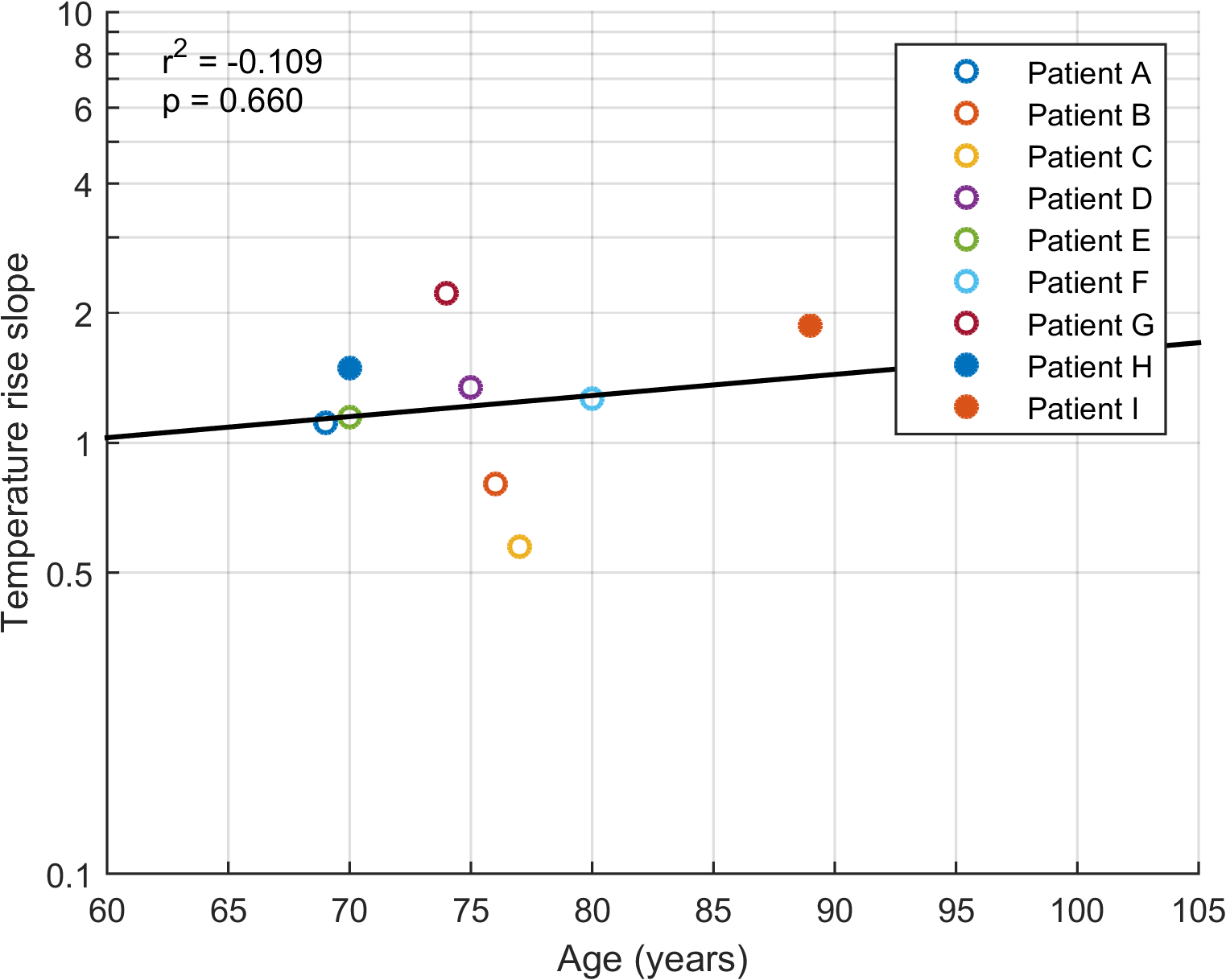

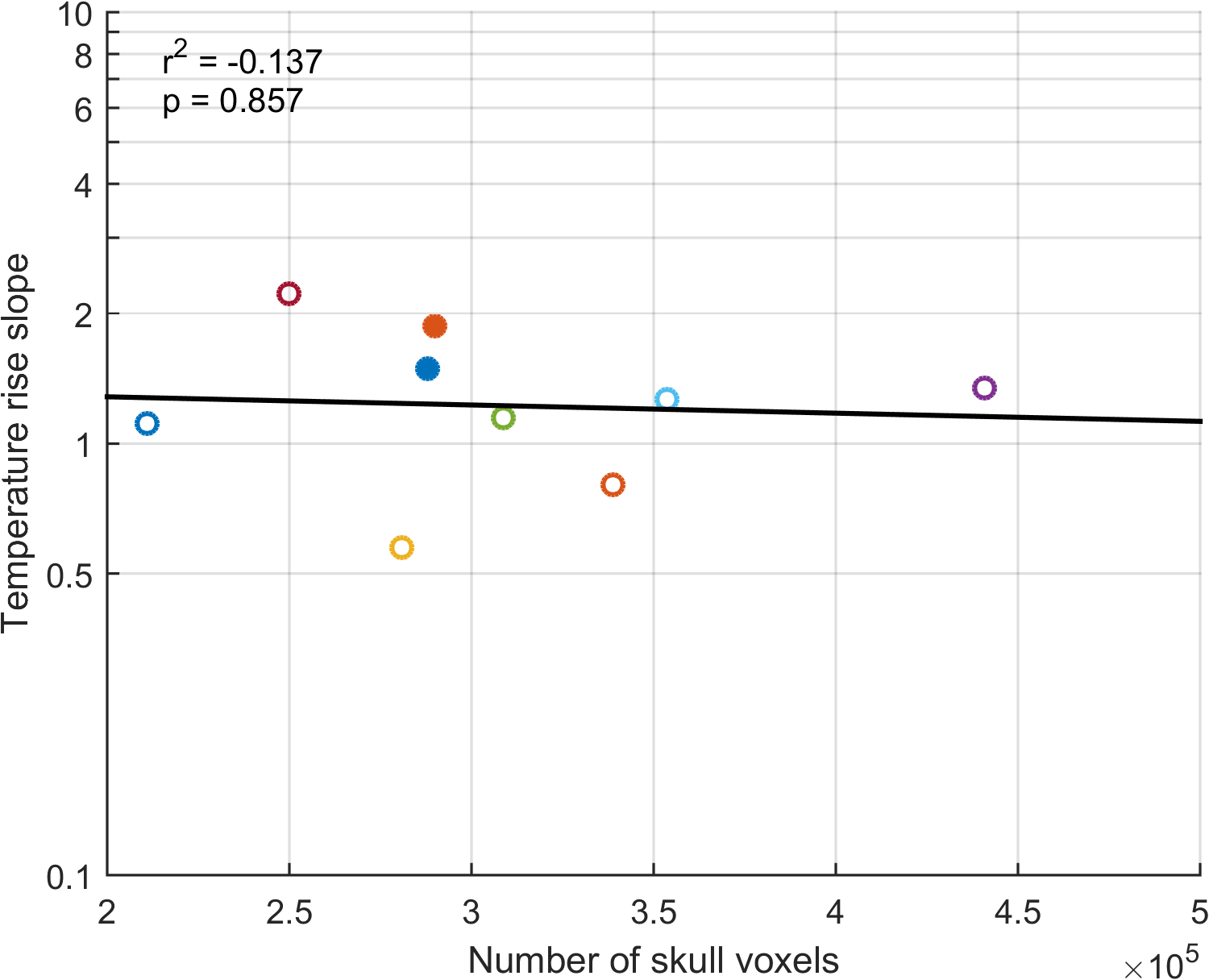


a) b)


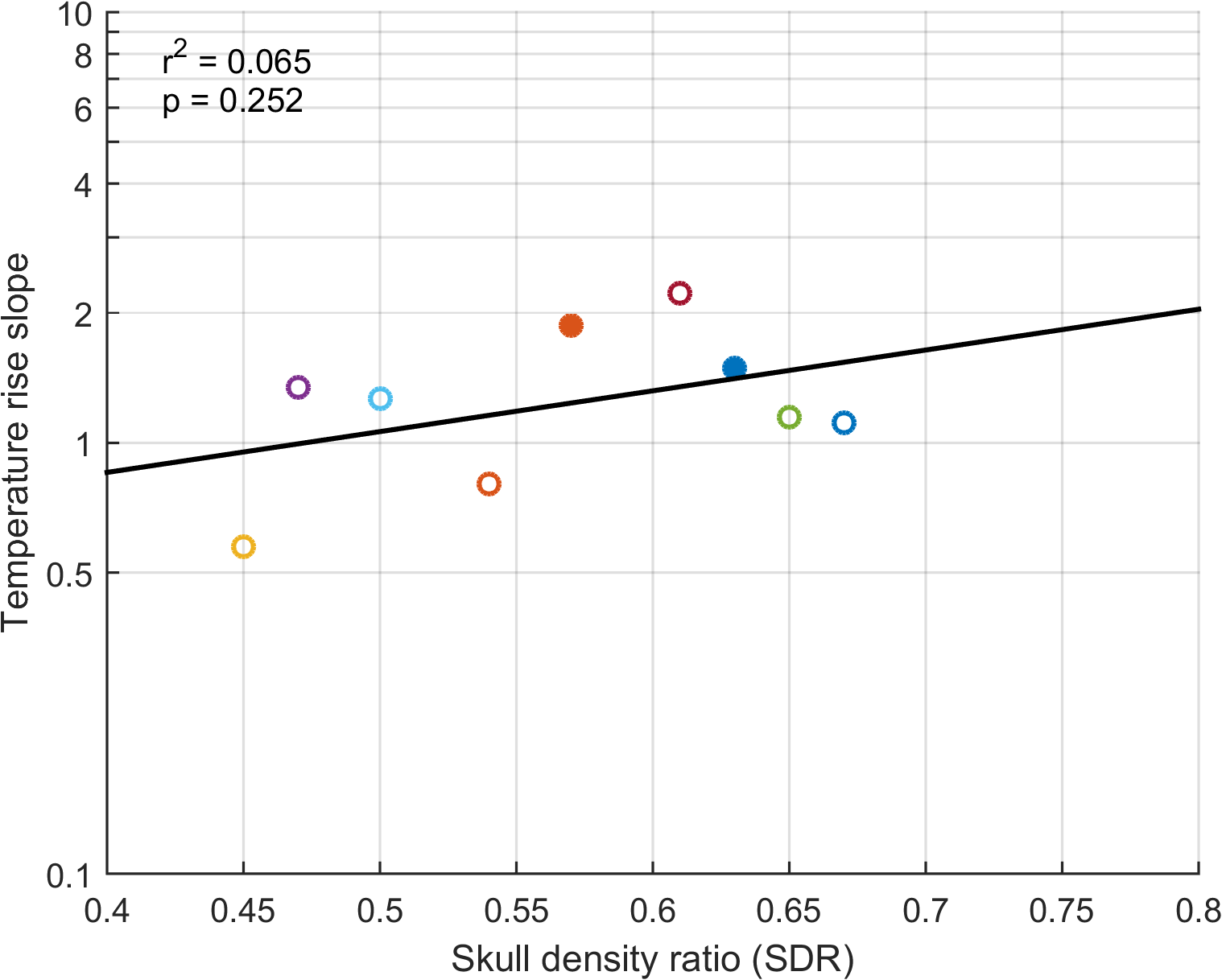


c)

**Supplementary Figure S2.** Temperature rise slope versus patient features. a) Age, b) size of skull, and c) skull density ratio did not predict whether simulation overestimated or underestimated temperature rise. Adjusted r^2^ values and p values are reported.


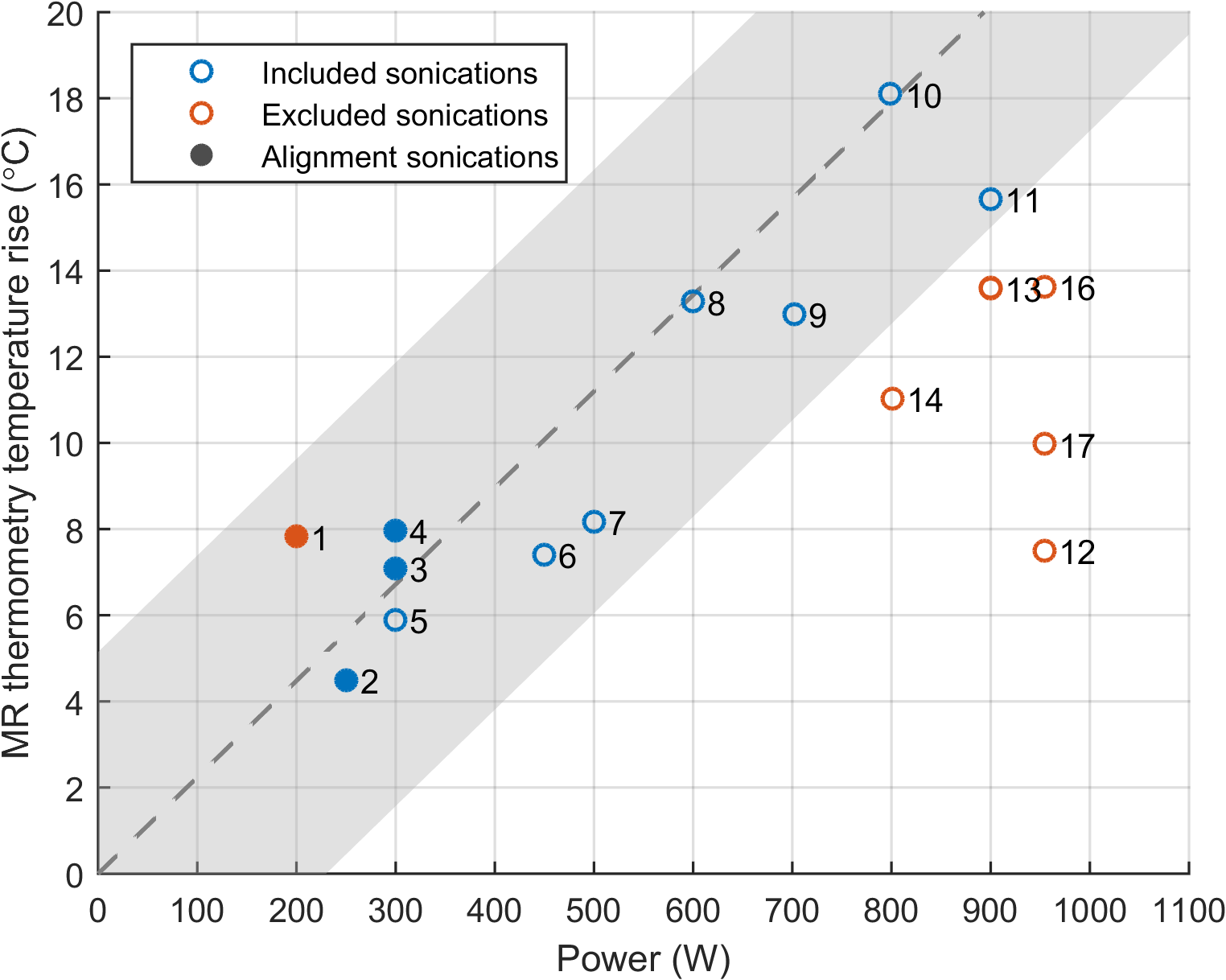

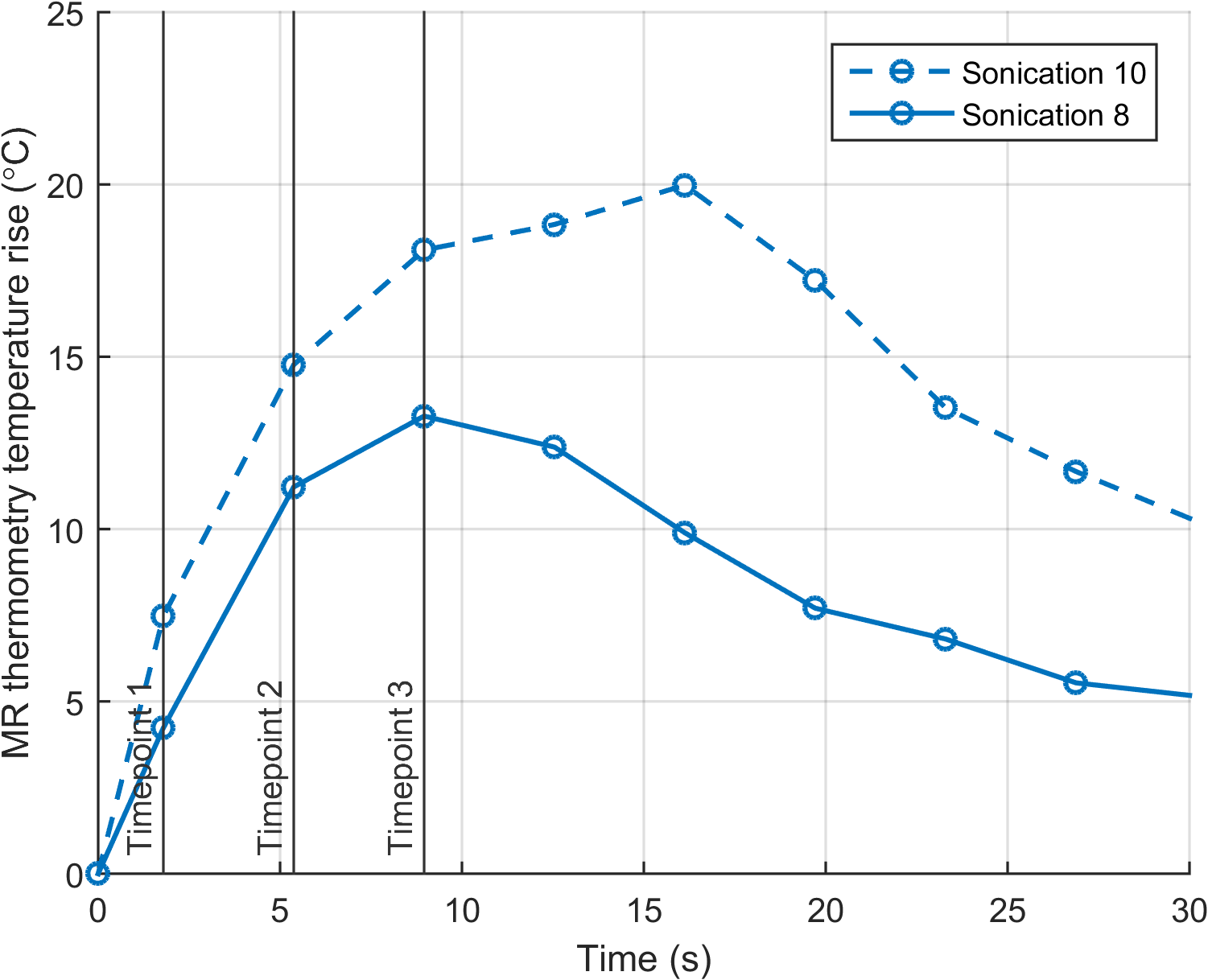


a) b)

**Supplementary Figure S3.** Method for selecting which sonications to simulate. a) Temperature rise versus power at the 3^rd^ MR thermometry image (timepoint 3) for sonications from the same treatment (patient D). Alignment sonications are denoted by filled markers. Sonications within the gray band were included for simulation. a) Two temperature rise time curves from the same treatment (patient D). The vertical lines indicate the time at which the center of k-space was acquired for the MR thermometry.


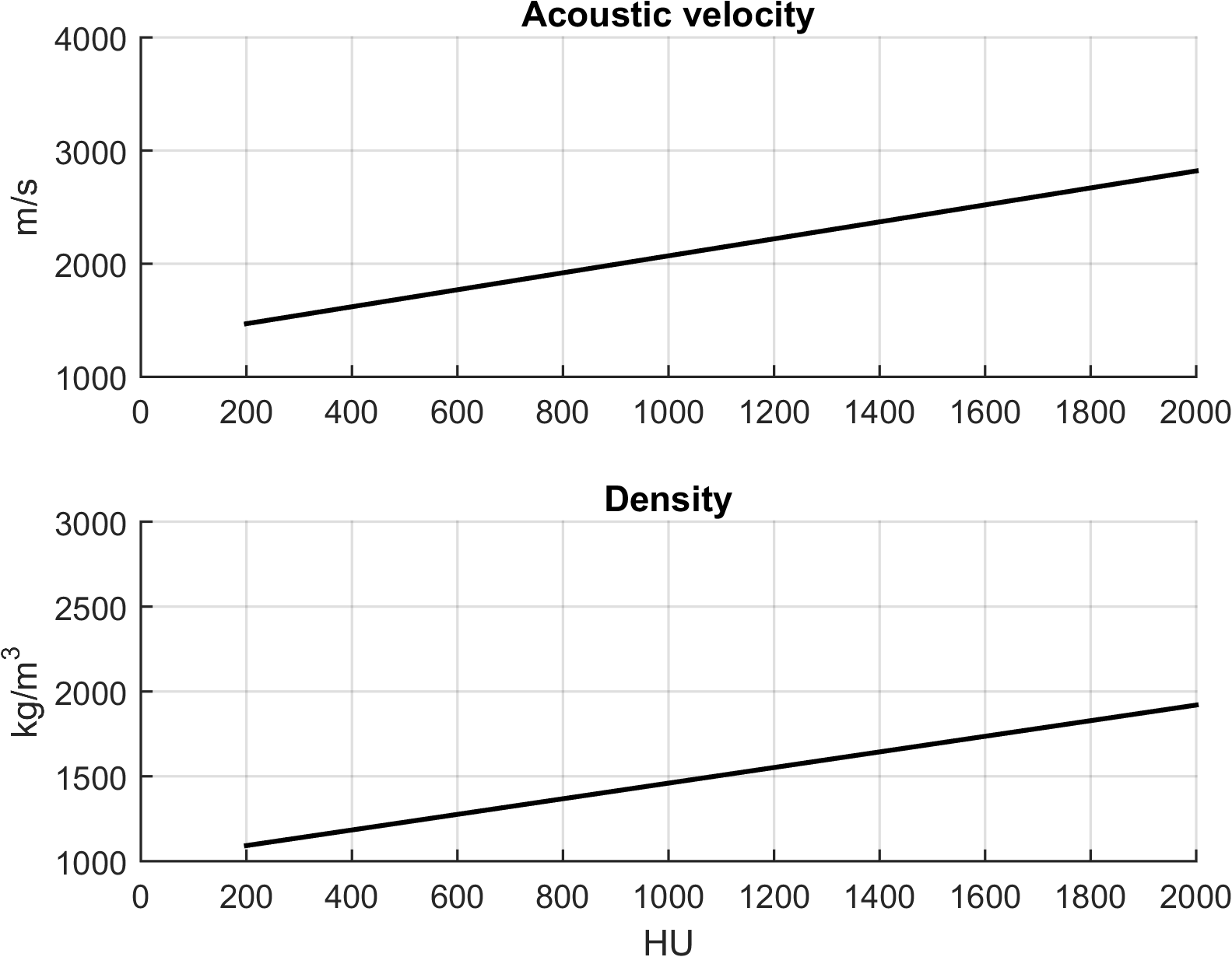

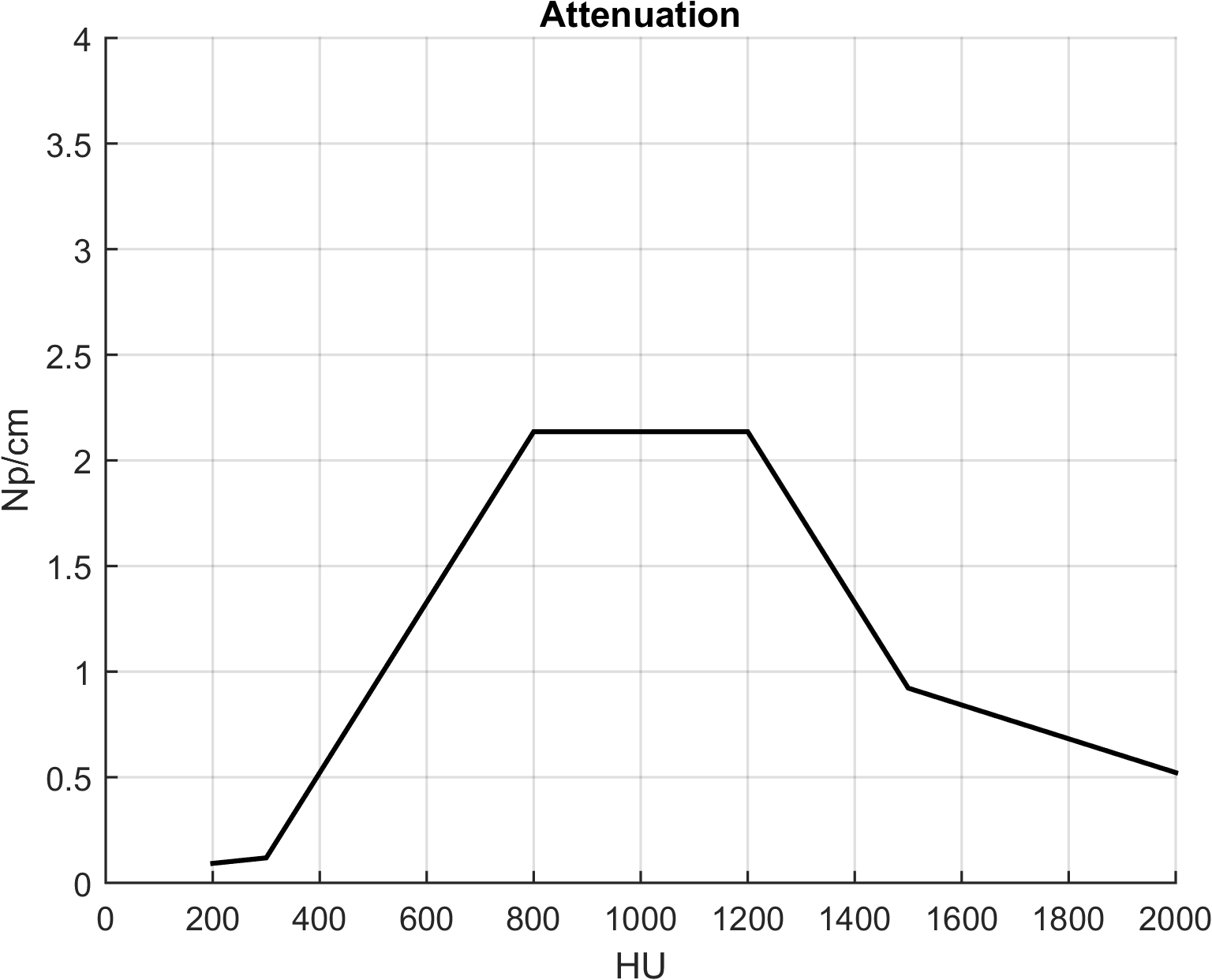


**Supplementary Figure S4**. Acoustic property relationships used to generate the skull model.


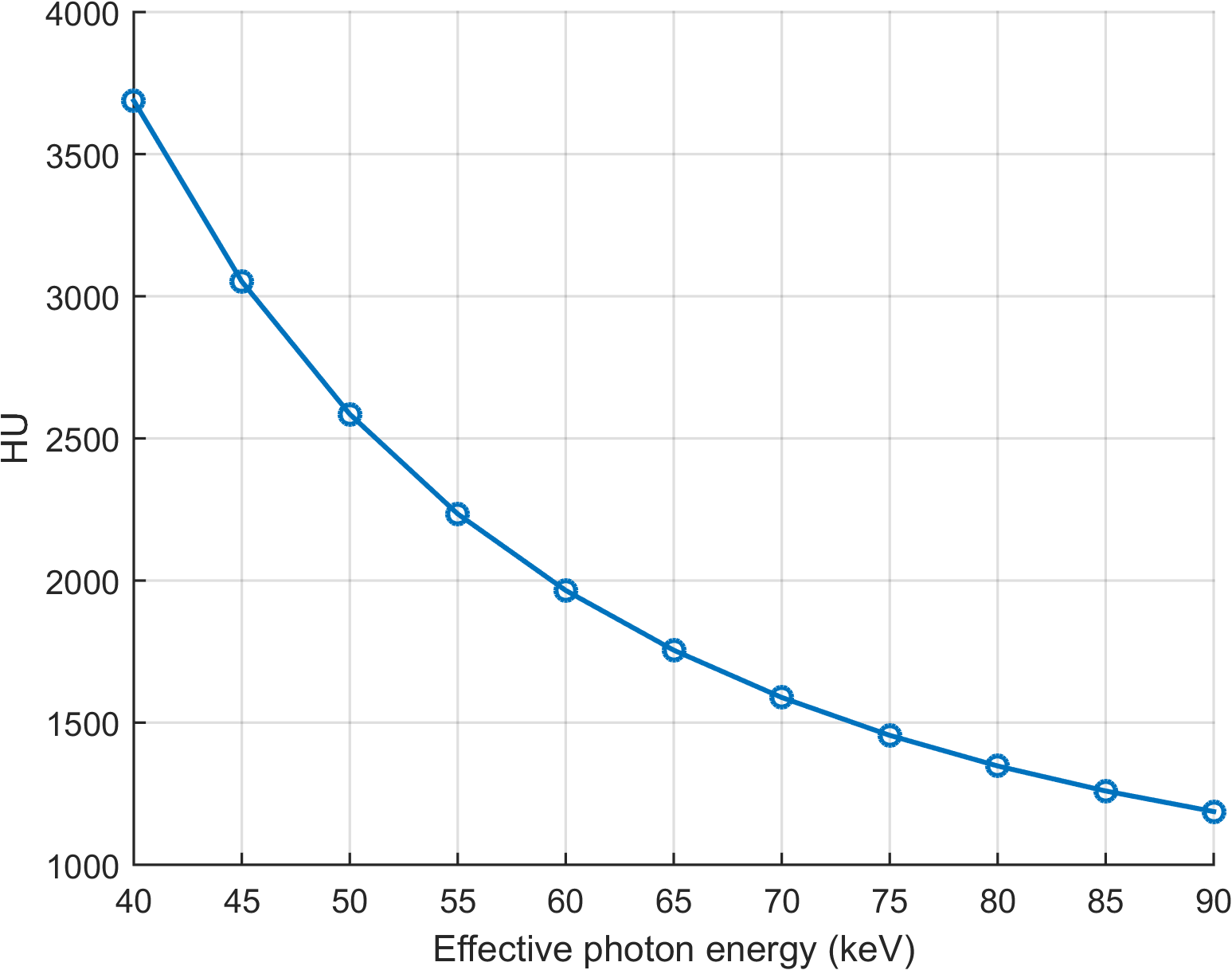


**Supplementary Figure S5.** Calculation of HU_bone_ as a function of effective photon energy. ρ_bone_ and mass attenuation coefficients for bone and water were referenced from the National Institute of Standards and Technology [^40^].


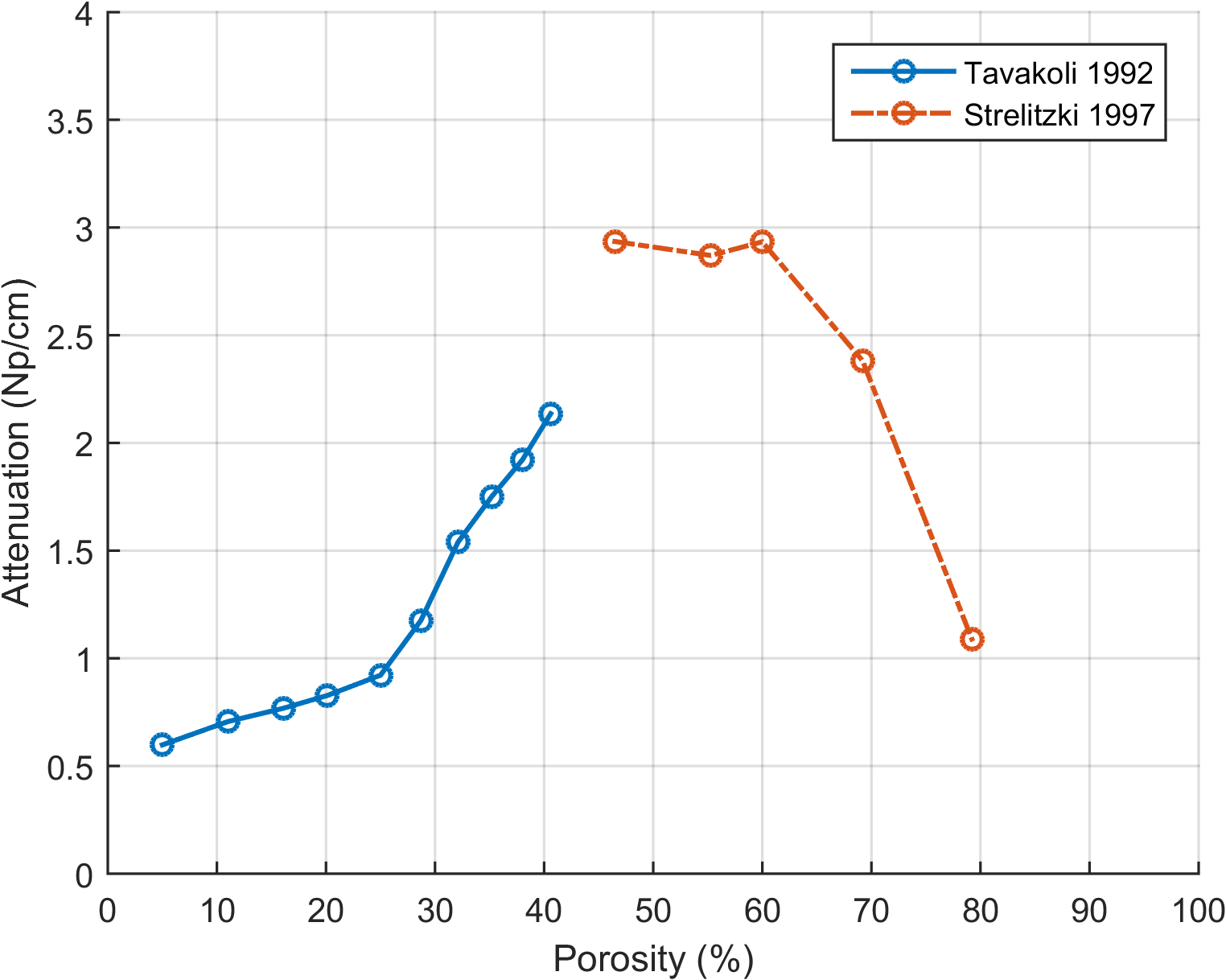

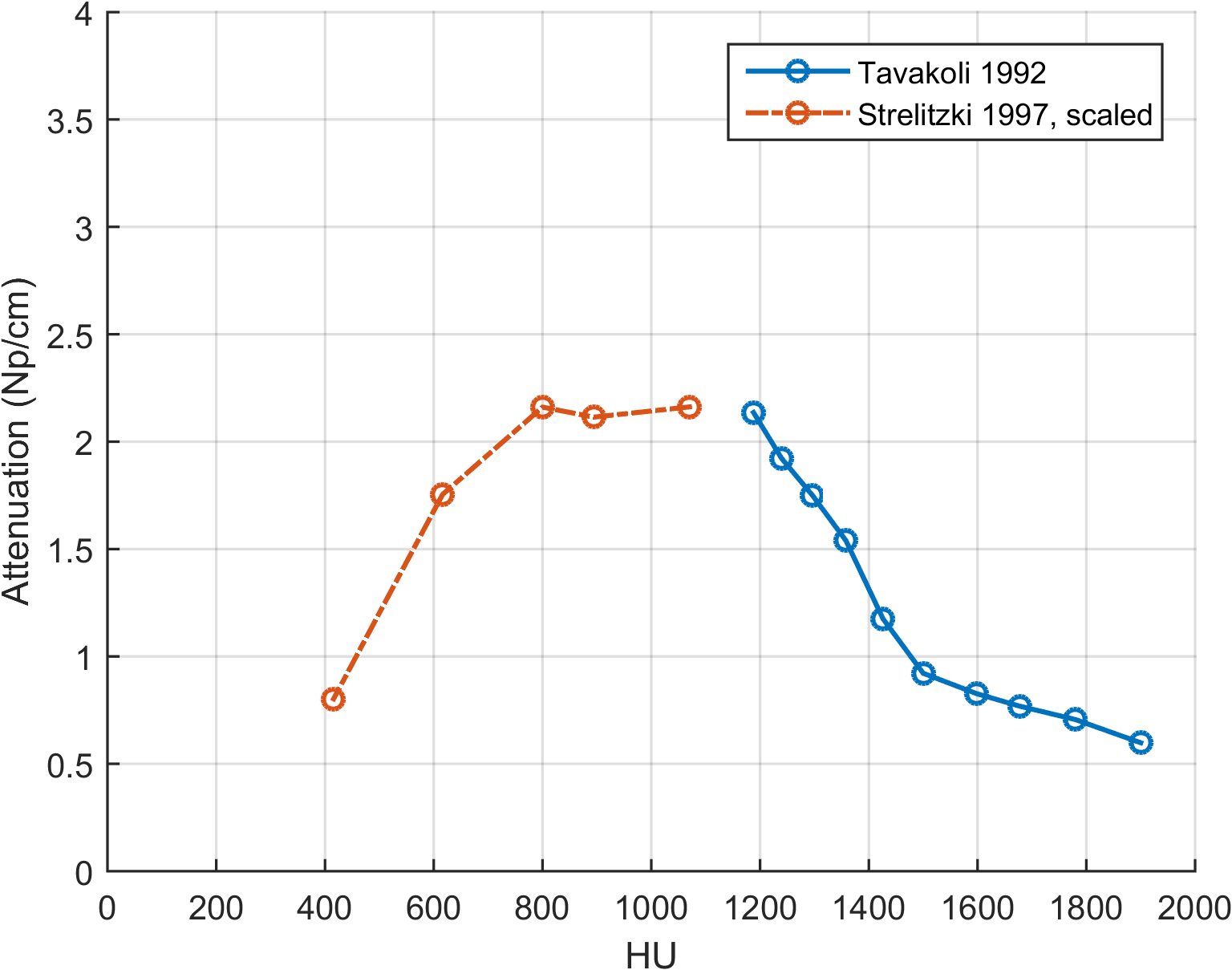


a) b)

**Supplementary Figure S6.** Derivation of the attenuation model for 680 kHz. a) Attenuation plotted against porosity. Tavakoli et al. [^41^] and Strelitzki et al. [^42^] report attenuation values for non-overlapping regions of porosity. b) Attenuation plotted against HU. Porosity of 0% was set at HU_bone_ = 2000. The Strelitzki et al. curve was scaled down to be consistent with the Tavakoli et al. curve.

|  | Single echo | Multi echo |
| --- | --- | --- |
| Echoes | 1 | 5 |
| TE | 12.772 ms | 3.288, 8.064, 12.840, 17.616, 22.392 ms |
| TR | 27.62 ms | 28 ms |
| Flip angle | 30° | 30° |
| Pixel bandwidth | 44.375 Hz | 278.984 Hz |
| Field of view | 280 mm | 280 mm |
| Slice thickness | 3 mm | 3 mm |
| Resolution | 1.094 x 2.188 mm | 1.094 x 2.188 mm |

**Supplementary Table S1.** MR thermometry parameters.

|  | Units | Relationship |
| --- | --- | --- |
| Acoustic velocity | m/s | c = 0.75 HU + 1320 |
| HU_bone_ | HU | 2000 |
| Bone fraction |  | f = HU / HU_bone_ |
| ρ_bone_ | kg/m^3^ | 1920 |
| ρ_water_ | kg/m^3^ | 1000 |
| Density | kg/m^3^ | ρ = ρ_bone_ f + ρ_water_ (1 – f) |
| Porosity | % | φ = 100 (1 – f) |
| Attenuation | Np/cm | Piecewise linear function:  α(φ=100) = 0.0412  α(φ=85) = 0.1183  α(φ=60) = 2.136  α(φ=40) = 2.136  α(φ=25) = 0.922  α(φ=0) = 0.522 |

**Supplementary Table S2**. Acoustic property relationships used to generate the skull model.

|  | Variable | Units | Brain tissue | Blood |
| --- | --- | --- | --- | --- |
| Base temperature | T | °C | 37 | 37 |
| Density | ρ | kg/m^3^ | 1046 | 1050 |
| Specific heat capacity | C | J/kg/°C | 3630 | 3617 |
| Conductivity | κ | W/m/°C | 0.51 |  |
| Perfusion rate | ω | ml/min/kg | 559 |  |
| Attenuation | α | Np/cm | 0.0412 |  |

**Supplementary Table S3.** Bioheat equation tissue properties. The tissue properties were referenced from the Foundation for Research on Information Technologies in Society (IT’IS) tissue properties database [^32^].
